# Supplementary material for: DDX41 resolves G-quadruplexes to maintain erythroid genome integrity and prevent cGAS-mediated cell death
Source: Nat Commun. 2025 Aug 5;16:7195. doi: 10.1038/s41467-025-62307-7 (PMC12325982; doi:10.1038/s41467-025-62307-7)
Supplement: Supplementary file 1 — Supplementary Information [file 41467_2025_62307_MOESM1_ESM.pdf]

Supplementary Information

Supplementary figures and figure legends

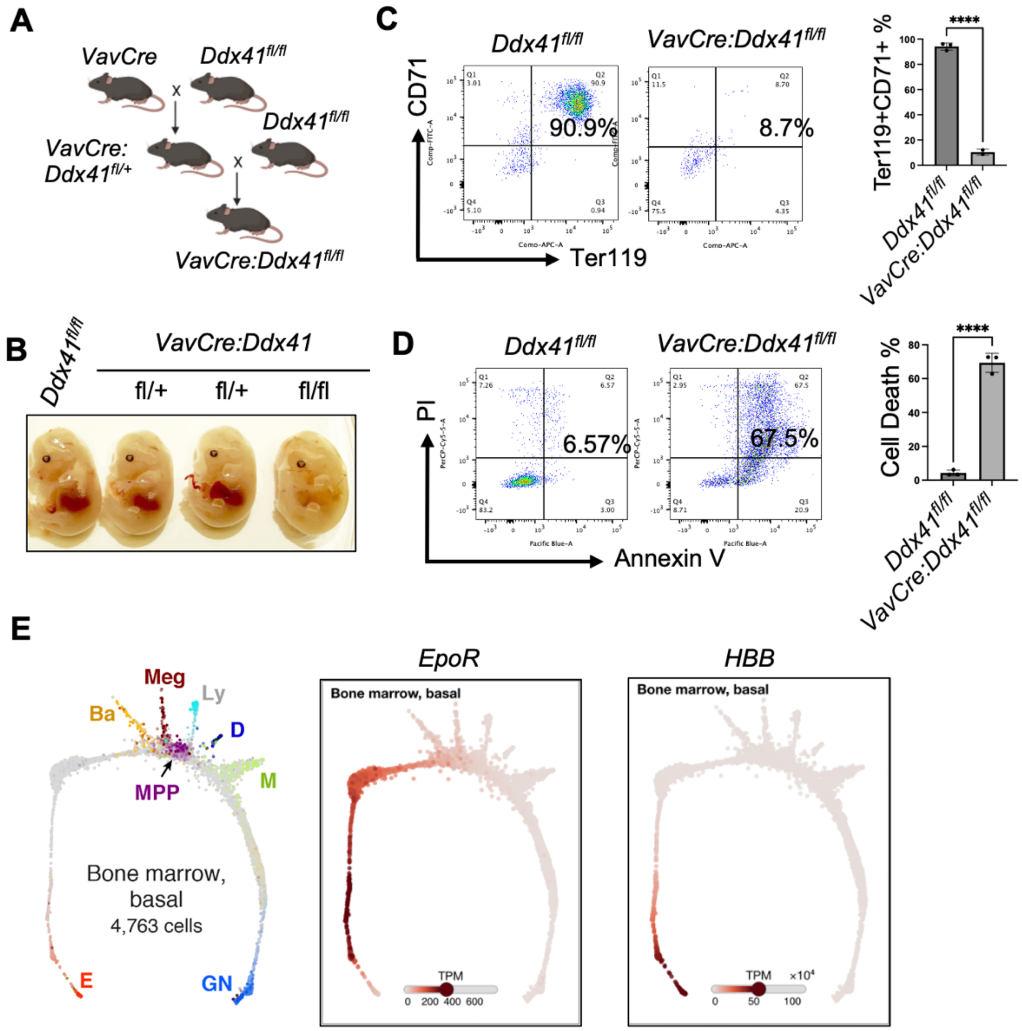

Supplementary Figure 1. Ddx41 is essential for hematopoiesis. (A) Schematic representation of the breeding strategy to generate hematopoietic-specific *Ddx41* knockout mice. Created in BioRender. Ji, P. (2025) https://BioRender.com/650mhm1. (B) Representative pictures of E14.5 embryos with the indicated genotypes. (C-D) Ter119 negative fetal liver cells from E14.5 embryos of the indicated mice were cultured in an Epo-containing medium for 48 hours. The differentiation status was evaluated by flow cytometry using CD71 and Ter119 markers. Cell viabilities were assessed using propidium iodide (PI) and Annexin V staining. Quantifications are on the right. (E) Expression patterns of EpoR and HBB during hematopoiesis. The left UMAP panel displays cells from various lineages and stages. The middle and right panels illustrate the expression pattern of EpoR and HBB, respectively. Figures were generated using the online tool accessible at https://kleintools.hms.harvard.edu/paper_websites/tusi_et_al/.

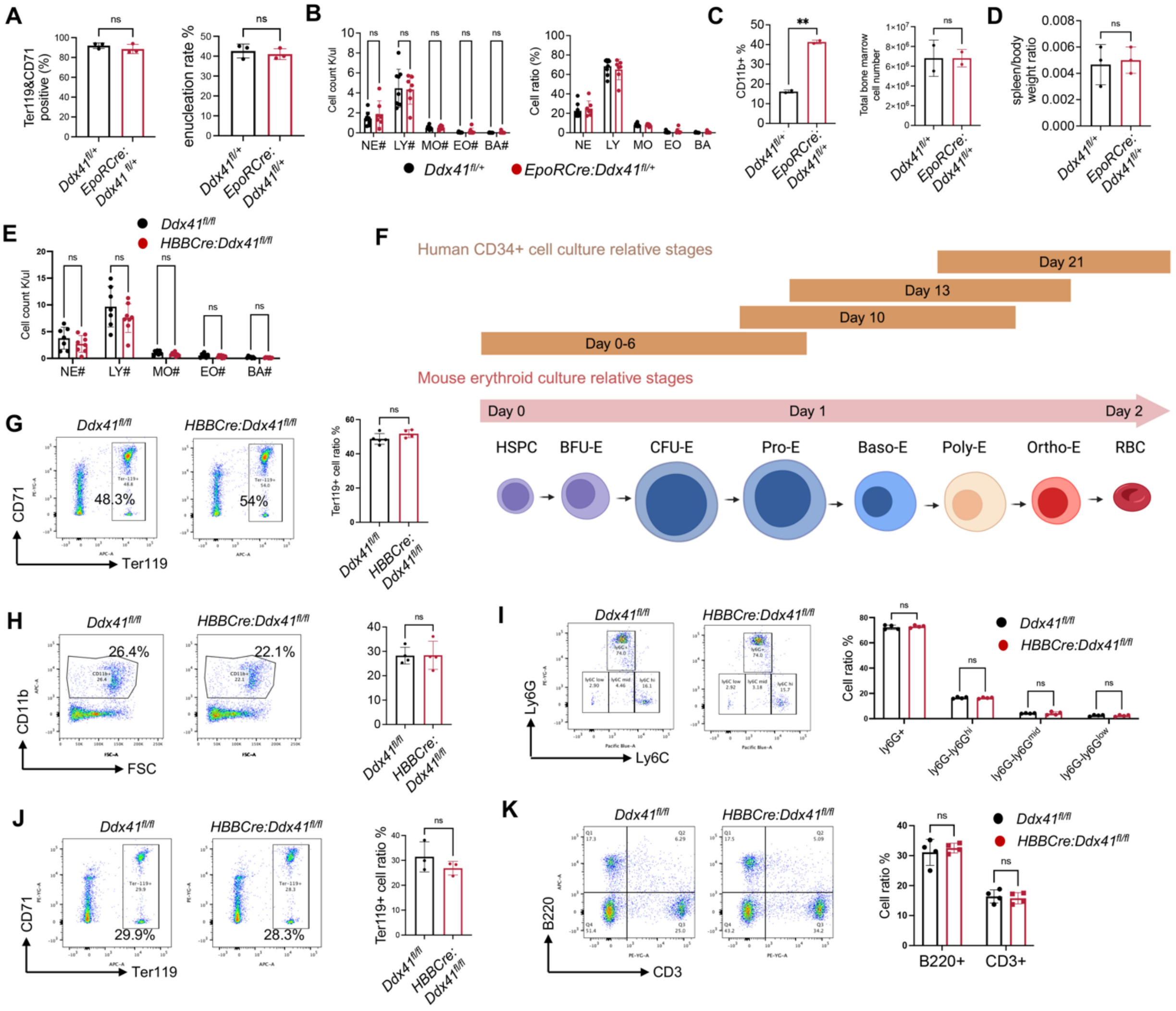

Supplementary Figure 2. Ddx41 is differentially required at different stages of terminal erythropoiesis. (A) Ter119-negative cells from the E13.5 fetal liver were purified from the indicated mice and cultured in Epo medium for 2 days. Cell differentiation and enucleation were measured by flow cytometry. (B) Leukocyte count of indicated mice at 2 months old. (C) Flow cytometry assay of bone marrow myeloid cells from mice in B using CD11b as a marker. Quantification of B and total bone marrow cell number is on the right. (D) Spleen/body weight ratio of indicated mice in A. (E) Leukocyte count of indicated mice at 2 months old. (F) Schematic diagram of the corresponding stages of erythropoiesis in the mouse and human in vitro culture systems. Created in BioRender. Ji, P. (2025) https://BioRender.com/9yztp7r. (G) Flow cytometry assay of bone marrow erythroid cells from mice in E using CD71 and Ter119 as markers. Quantification is on the right. (H-K) Flow cytometry assays of bone marrow myeloid (H), bone marrow myelomonocytic (I), bone marrow erythroid (J), and spleen lymphoid (K) populations from mice in E. Quantifications are on the right. At least three independent samples were used in each experiment. P-values were determined with 2 tailed t tests. *p<0.05, **p<0.01, ***p<0.001, ****p<0.0001.

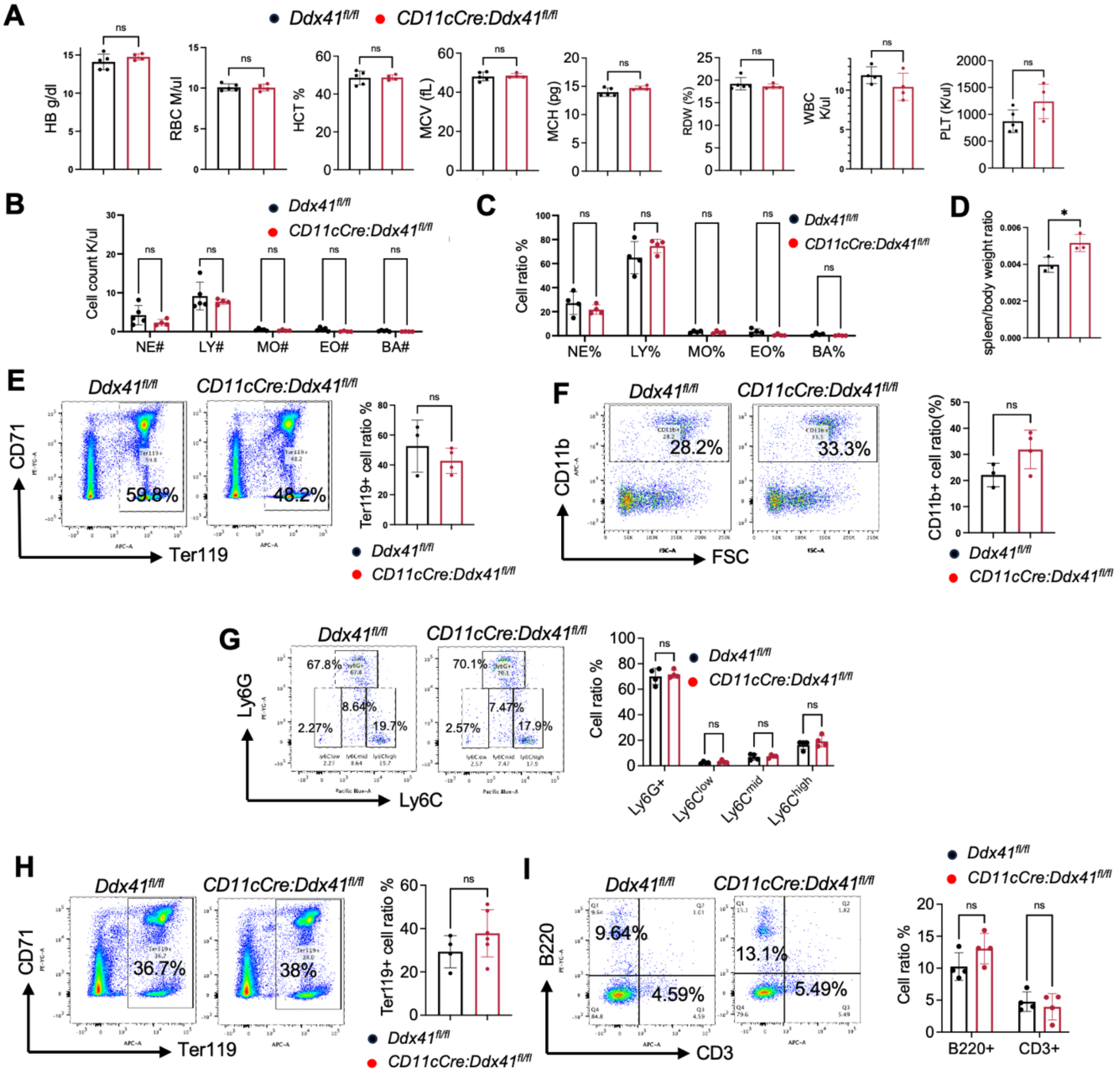

Supplementary Figure 3. Ddx41 is dispensable for dendritic cell differentiation. (A) Complete blood count of indicated mice at 2 months old. (B-C) Leukocyte absolute number (B) and percentage (C) of mice from A. (D) Spleen/body weight ratio of indicated mice in A. (E-I) Flow cytometry assays of bone marrow erythroid (E), bone marrow myeloid (F), bone marrow myelomonocytic (G), spleen erythroid (H), and spleen lymphoid (I) populations from mice in A. Quantifications are on the right. Each panel represents data obtained from a minimum of three independent samples. P-values were determined with 2 tailed t tests. ns: non-significant, *p<0.05, **p<0.01, ***p<0.001, ****p<0.0001.

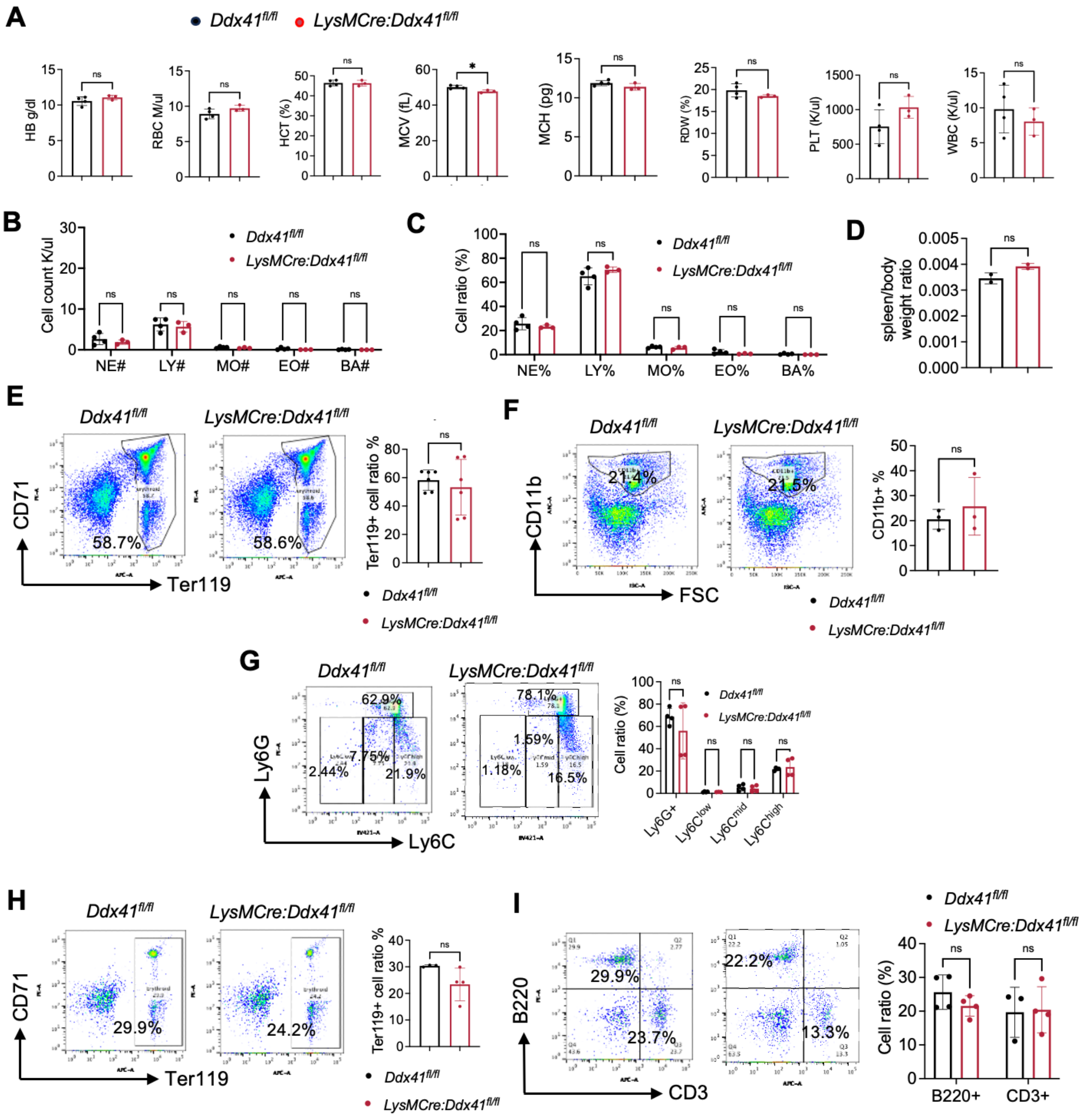

Supplementary Figure 4. **Ddx41 is dispensable for monocytic cell differentiation.** (A) Complete blood count of indicated mice at 2 months old. (B-C) Leukocyte absolute number (B) and percentage (C) of mice from A. (D) Spleen/body weight ratio of indicated mice in A. (E-I) Flow cytometry assays of bone marrow erythroid (E), bone marrow myeloid (F), bone marrow myelomonocytic (G), spleen erythroid (H), and spleen lymphoid (I) populations from mice in A. Quantifications are on the right. Each panel represents data obtained from a minimum of three independent samples. P-values were determined with 2 tailed t tests. ns: non-significant, *p<0.05, **p<0.01, ***p<0.001, ****p<0.0001.

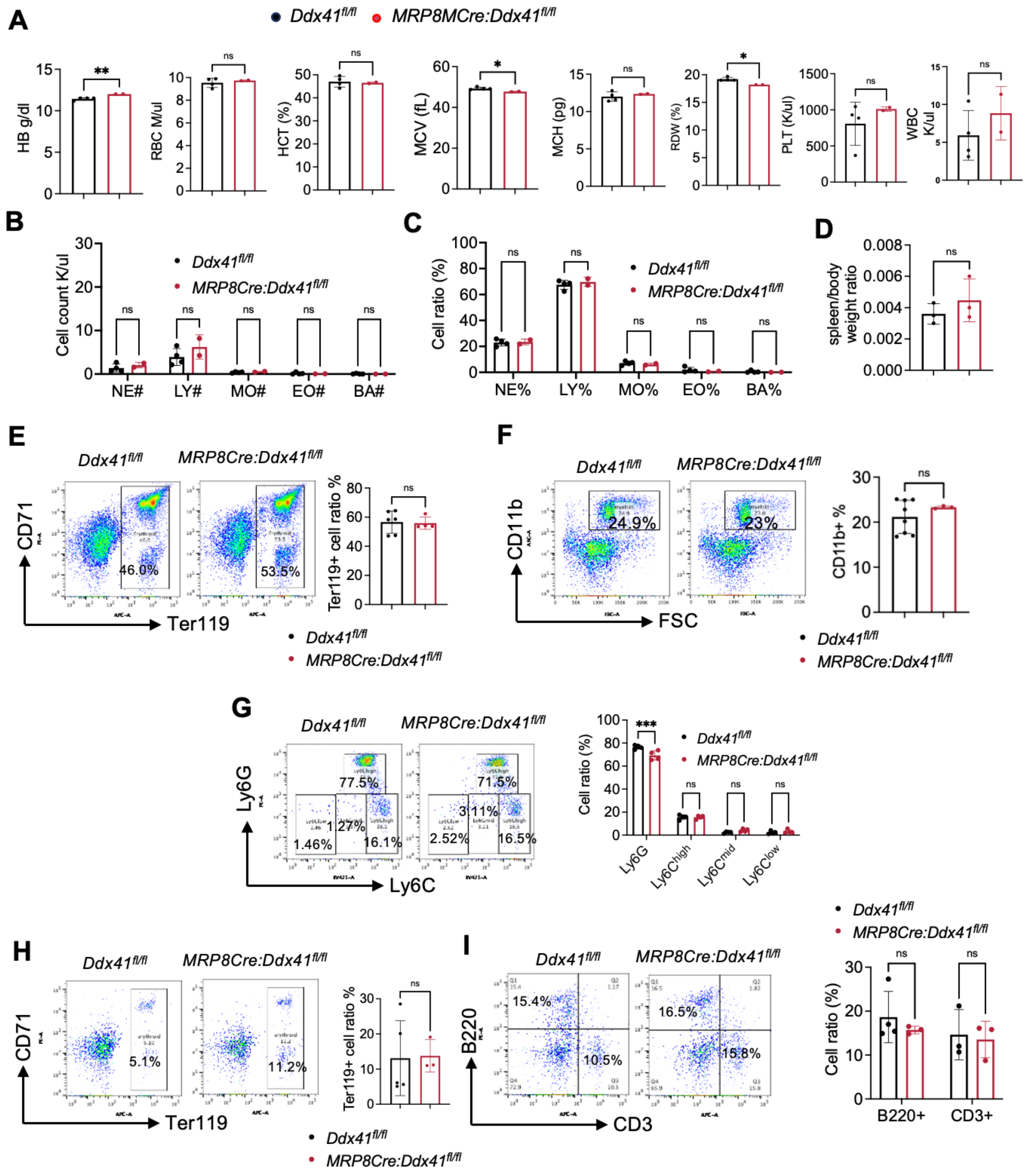

Supplementary Figure 5. Ddx41 is dispensable for myeloid cell differentiation. (A) Complete blood count of indicated mice at 2 months old. (B-C) Leukocyte absolute number (B) and percentage (C) of mice from A. (D) Spleen/body weight ratio of indicated mice in A. (E-I) Flow cytometry assays of bone marrow erythroid (E), bone marrow myeloid (F), bone marrow myelomonocytic (G), spleen erythroid (H), and spleen lymphoid (I) populations from mice in A. Quantifications are on the right. Each panel represents data obtained from a minimum of three independent samples. P-values were determined with 2 tailed t tests. ns: non-significant, *p<0.05, **p<0.01, ***p<0.001, ****p<0.0001.

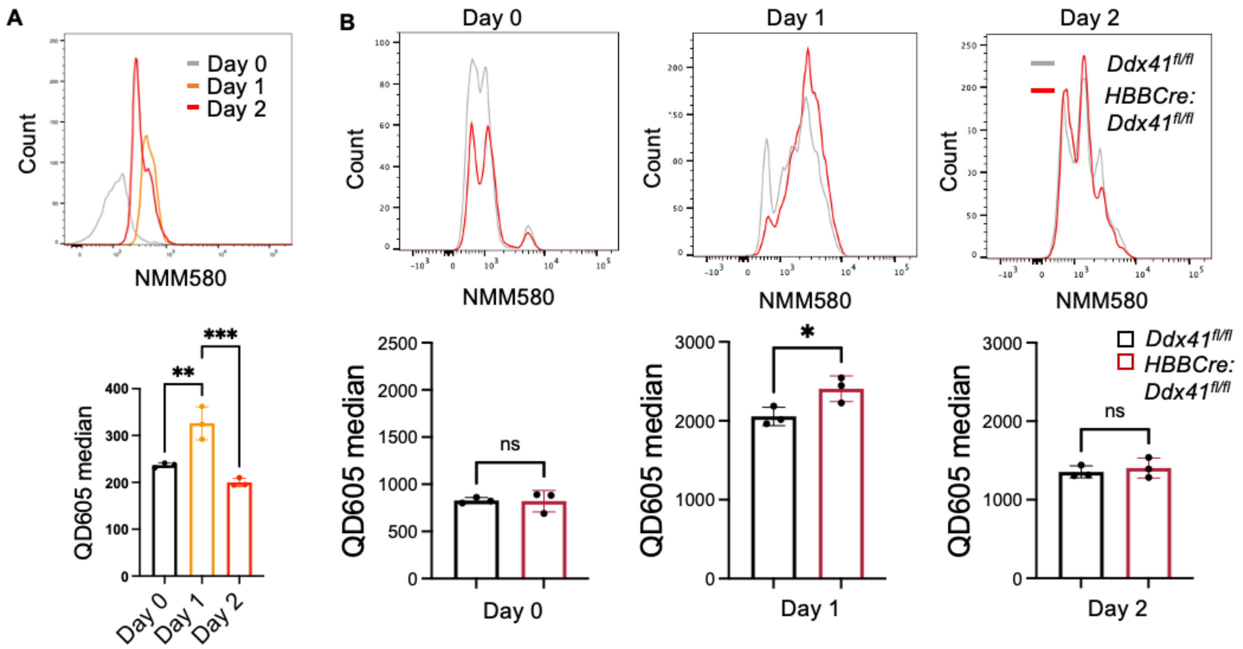

Supplementary Figure 6. G-quadruplex level changes during erythropoiesis. (A) Bone marrow lineage-negative cells were cultured in Epo medium for 2 days. G4 levels were tested on different days using flow cytometry by NMM580 (Frontier Scientific, Cas 142234-85-3). Quantification is shown below. (B) Bone marrow lineage negative cells from the indicated mice were cultured in Epo medium for 2 days. G4 levels on different days were measured by flow cytometry using NMM580. Quantification is shown below each histogram. The comparison among multiple groups was evaluated with 1-way ANOVA tests. The comparison between two groups was evaluated with 2 tailed t test. ns: non-significant. *p<0.05, **p<0.01, ***p<0.001.

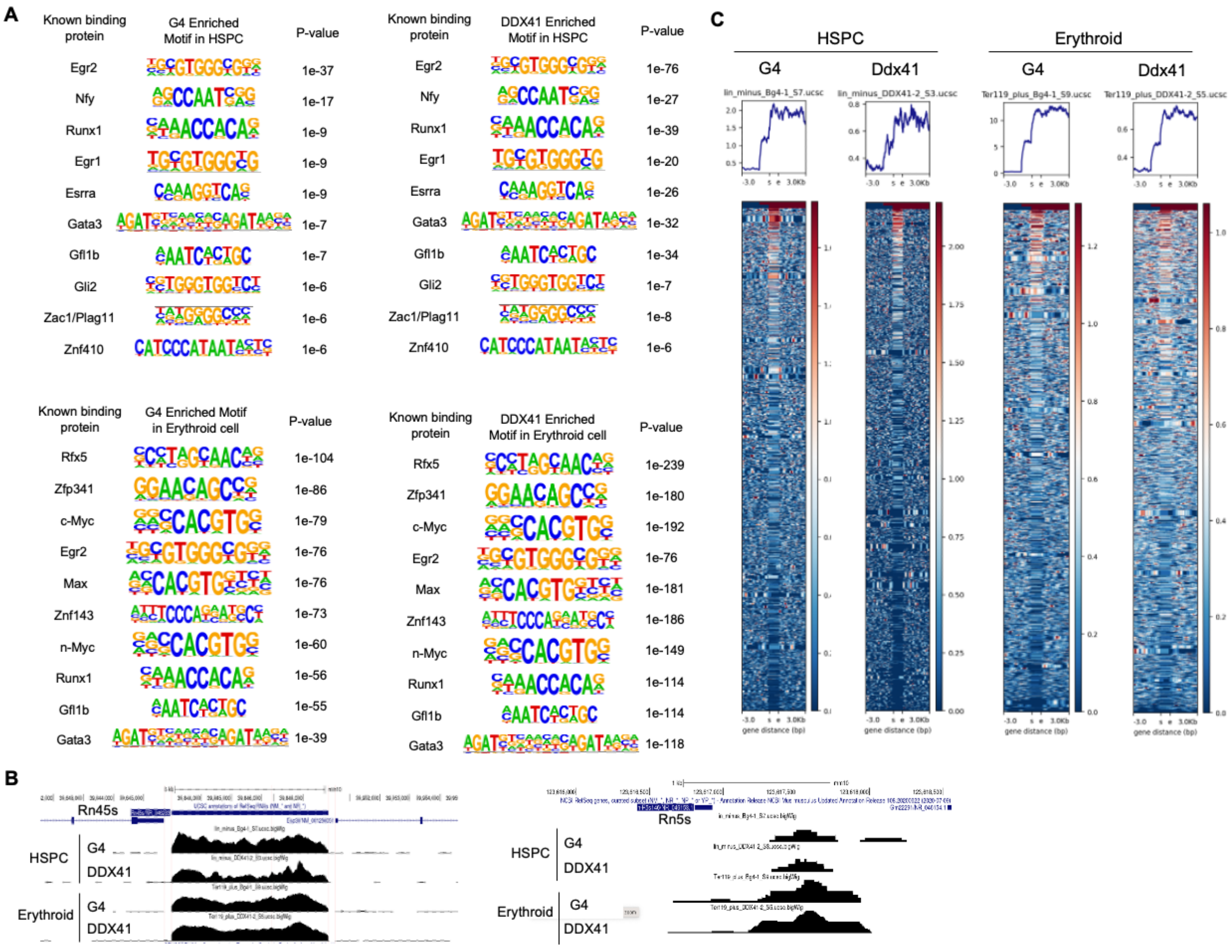

Supplementary Figure 7. Ddx41 co-distributes with G4 at the erythroid genome level. (A) Motif enrichment analysis of co-distributed G4 and Ddx41 sites in mouse HSPC and erythroid cells. Motif enrichment analysis was conducted using the Homer Motif analysis tool, with no GC weight applied. (B-C) Enrichment of G4 and Ddx41 binding sites in rDNAs in HSPC and erythroid cells. Panel B displays exemplary data with G4 and Ddx41 binding peaks enriched in Rn45s and Rn5s genes. Panel C presents a heat map illustrating the localization of G4 and Ddx41 within rDNAs. The rDNA sequences were retrieved from the UCSC genome browser using the selection criteria of matching gene type '*rRNA' from the mm10 (VM25) assembly.

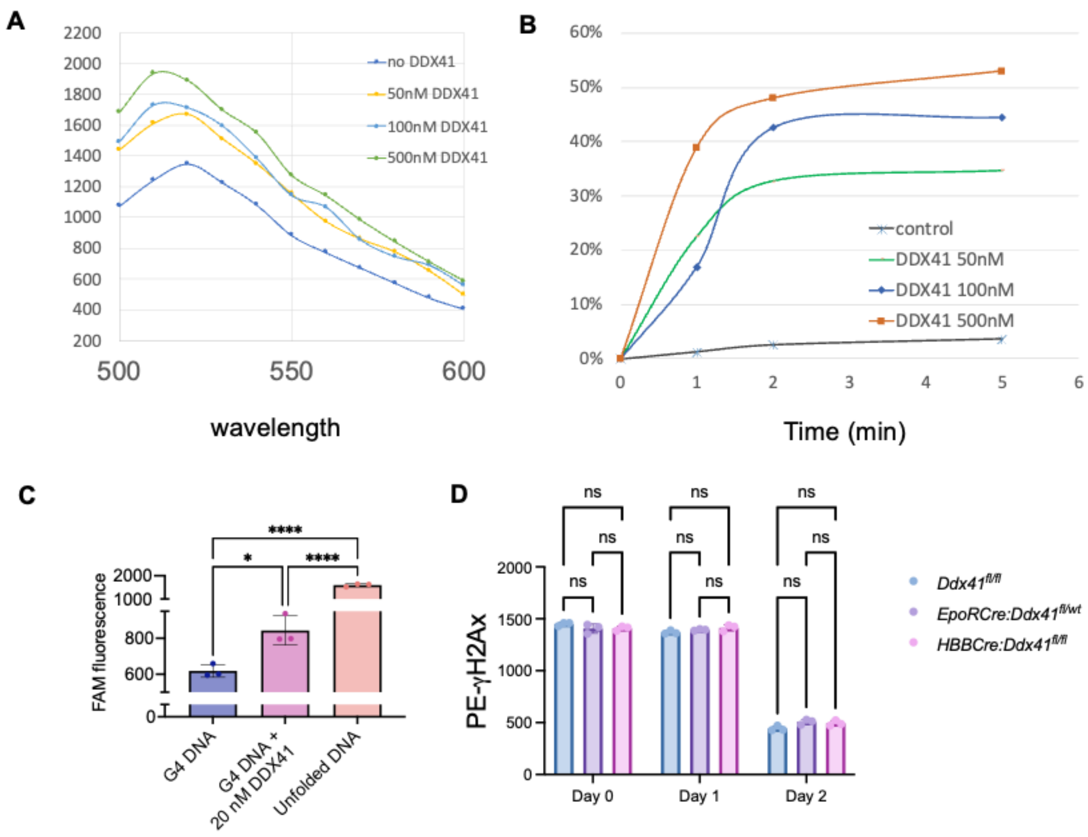

Supplementary Figure 8. DDX41 dose-dependently dissolves G4. (A) Dose-dependent increase of FAM signal with increased concentration of recombinant human DDX41 protein. (B) Time course of DDX41-mediated G4 dissolving activity. (C) Statistical analysis of FAM signals with 20 nM DDX41 compared to the G4 DNA only group and the unfolded DNA group. (D) Lineage-negative cells from the bone marrow of the indicated mice were purified and cultured in Epo medium for 2 days. The levels of γ-H2AX were measured and quantified using flow cytometry. The comparison among multiple groups was evaluated with 1-way ANOVA tests. *$p<0.05$, ****$p<0.0001$, ns: non-significant.

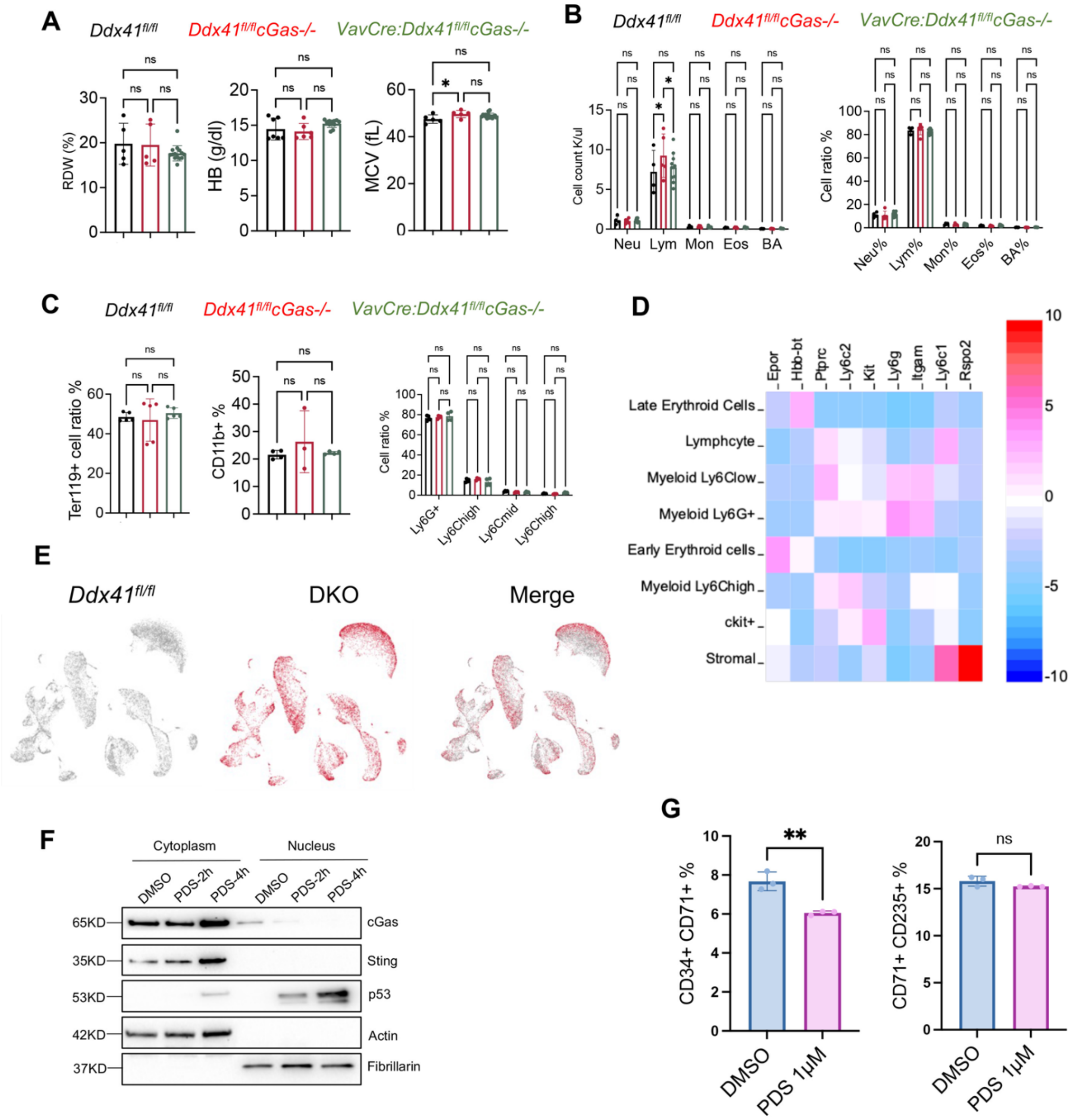

Supplementary Figure 9. cGas deficiency rescues the embryonic lethality of hematopoietic specific Ddx41 knockout mice. (A) Red cell indices of the indicated mice at 2 months old. (B) Leukocyte absolute number (left) and percentage (right) of mice from A. (C) Quantification of flow cytometry assays of bone marrow erythroid (left), myeloid (middle), and myelomonocytic (right) from mice in A. (D) Specific marker genes in the identified cell clusters in the scRNA sequencing data. (E) Uniform Manifold Approximation and Projection (UMAP) plots showing the distribution and overlapping of different cell populations in the bone marrow of indicated mice from A. (F) Western blotting assays of indicated proteins in the cytoplasmic and nuclear fractions of bone marrow erythroid cells treated with DMSO or PDS (1 μM) for the indicated amount of time. Lineage-negative cells from the bone marrow of wild-type mice were cultured in Epo medium for 1 day, followed by the treatment. (G) Human bone marrow organoids were treated with PDS for 24 hours followed by flow cytometry assays of the indicated cell types. All the error bars represent the SEM of the mean. The comparison among multiple groups was evaluated with 1-way ANOVA tests. The comparison between two groups was evaluated with 2 tailed t test. ns: non-significant. *p<0.05, **p<0.01.

Supplementary Table 1

Lineage-specific Cre system	Expression lineage	Earliest knockout stage	Viable homozygous mutant?	Homozygous fetuses acquired or not?
Vav-Cre	HSCs and HSPCs	HSCs (~E13.5)	No	Yes
EpoR-Cre	Erythrocytes	Primitive erythroblasts, early terminal erythroblasts	No	Yes
HBB-Cre	Erythrocytes	Late terminal erythroblasts	Yes	Yes
CD11c-Cre	Dendritic cells (DCs)	Pre-cDCs, pre-pDCs	Yes	Yes
MRP8-Cre	Granulocytes, granulocyte/macrophage progenitors (GMPs)	Myelocyte stage	Yes	Yes
LysM-Cre	Monocytes, mature macrophages, and granulocytes	Immature monocytes	Yes	Yes
