# Supplementary material for: DDX41 resolves G-quadruplexes to maintain erythroid genome integrity and prevent cGAS-mediated cell death
Source: Nat Commun. 2025 Aug 5;16:7195. doi: 10.1038/s41467-025-62307-7 (PMC12325982; doi:10.1038/s41467-025-62307-7)
Supplement: Supplementary file 2 — Description of Additional Supplementary Files [file 41467_2025_62307_MOESM2_ESM.pdf]

Description of Additional Supplementary Files

File Name: Supplementary Data 1

Description: List of genes with altered expression in late erythroid cells in wild type and DKO mice.
