# Supplementary material for: DDX41 resolves G-quadruplexes to maintain erythroid genome integrity and prevent cGAS-mediated cell death
Source: Nat Commun. 2025 Aug 5;16:7195. doi: 10.1038/s41467-025-62307-7 (PMC12325982; doi:10.1038/s41467-025-62307-7)
Supplement: Supplementary file 4 — Reporting Summary [file 41467_2025_62307_MOESM4_ESM.pdf]

nature portfolio

Corresponding author(s): Peng Ji

Last updated by author(s): 06/26/2025

Reporting Summary

Nature Portfolio wishes to improve the reproducibility of the work that we publish. This form provides structure for consistency and transparency in reporting. For further information on Nature Portfolio policies, see our Editorial Policies and the Editorial Policy Checklist.

Statistics

For all statistical analyses, confirm that the following items are present in the figure legend, table legend, main text, or Methods section.

n/a	Confirmed	
☐	☒	The exact sample size (*n*) for each experimental group/condition, given as a discrete number and unit of measurement
☐	☒	A statement on whether measurements were taken from distinct samples or whether the same sample was measured repeatedly
☐	☒	The statistical test(s) used AND whether they are one- or two-sided<br>*Only common tests should be described solely by name; describe more complex techniques in the Methods section.*
☐	☒	A description of all covariates tested
☒	☐	A description of any assumptions or corrections, such as tests of normality and adjustment for multiple comparisons
☐	☒	A full description of the statistical parameters including central tendency (e.g. means) or other basic estimates (e.g. regression coefficient) AND variation (e.g. standard deviation) or associated estimates of uncertainty (e.g. confidence intervals)
☐	☒	For null hypothesis testing, the test statistic (e.g. *F*, *t*, *r*) with confidence intervals, effect sizes, degrees of freedom and *P* value noted<br>*Give P values as exact values whenever suitable.*
☒	☐	For Bayesian analysis, information on the choice of priors and Markov chain Monte Carlo settings
☒	☐	For hierarchical and complex designs, identification of the appropriate level for tests and full reporting of outcomes
☒	☐	Estimates of effect sizes (e.g. Cohen's *d*, Pearson's *r*), indicating how they were calculated

Our web collection on statistics for biologists contains articles on many of the points above.

Software and code

Policy information about availability of computer code

Data collection	All data were generated by the authors.
Data analysis	scRNA-seq data was analyzed using Cellranger and visualized by cloupe browser. Cut&run data were analyzed using bedtools.

For manuscripts utilizing custom algorithms or software that are central to the research but not yet described in published literature, software must be made available to editors and reviewers. We strongly encourage code deposition in a community repository (e.g. GitHub). See the Nature Portfolio guidelines for submitting code & software for further information.

Data

Policy information about availability of data

All manuscripts must include a data availability statement. This statement should provide the following information, where applicable:

- Accession codes, unique identifiers, or web links for publicly available datasets
- A description of any restrictions on data availability
- For clinical datasets or third party data, please ensure that the statement adheres to our policy

The sequencing data are available from the Gene Expression Omnibus database under accession code GSE254144 and GSE254143

Research involving human participants, their data, or biological material

Policy information about studies with human participants or human data. See also policy information about sex, gender (identity/presentation), and sexual orientation and race, ethnicity and racism.

Reporting on sex and gender	Human participants or human data are not involved. All human samples were de-identified and exempt from human subject research by NIH guideline.
Reporting on race, ethnicity, or other socially relevant groupings	NA
Population characteristics	NA
Recruitment	NA
Ethics oversight	NA

Note that full information on the approval of the study protocol must also be provided in the manuscript.

Field-specific reporting

Please select the one below that is the best fit for your research. If you are not sure, read the appropriate sections before making your selection.

☒ Life sciences ☐ Behavioural & social sciences ☐ Ecological, evolutionary & environmental sciences

For a reference copy of the document with all sections, see nature.com/documents/nr-reporting-summary-flat.pdf

Life sciences study design

All studies must disclose on these points even when the disclosure is negative.

Sample size	Sample sizes corresponding to all data are reported in the manuscript. No formal statistical power calculations were performed to predetermine sample sizes. Instead, sample sizes for all in vivo and in vitro experiments were based on prior studies using similar models and experimental designs in the field, as well as our own previously published work. For mouse experiments, we typically used 3–6 animals per group, which has proven sufficient to detect biologically meaningful differences in erythropoiesis and hematopoietic phenotypes. For cell-based assays, experiments were performed in biological triplicates and repeated independently to ensure reproducibility. These sample sizes are consistent with established standards in hematology and molecular biology research and were sufficient to achieve statistical significance where appropriate, as indicated in the figure legends. All statistical analyses were conducted post hoc using appropriate tests, as detailed in the Methods section. For all experiments, allocation of samples or animals into experimental groups was not randomized. Experimental grouping was based on defined genotypes, treatments, or time points relevant to the biological questions being addressed.
Data exclusions	No exclusion
Replication	All experiments were confirmed to be replicatable. Each experiment was repeated at least 3 times.
Randomization	Mice were randomly selected for experiment. Littermates from the same parents but with different genotypes were used as controls.
Blinding	Researchers were intentionally blinded from group names (experimental or control) during statistical analysis.

Reporting for specific materials, systems and methods

We require information from authors about some types of materials, experimental systems and methods used in many studies. Here, indicate whether each material, system or method listed is relevant to your study. If you are not sure if a list item applies to your research, read the appropriate section before selecting a response.

Materials & experimental systems

n/a	Involved in the study
☐	☒ Antibodies
☐	☒ Eukaryotic cell lines
☒	☐ Palaeontology and archaeology
☐	☒ Animals and other organisms
☒	☐ Clinical data
☒	☐ Dual use research of concern
☒	☐ Plants

Methods

n/a	Involved in the study
☒	☐ ChIP-seq
☐	☒ Flow cytometry
☒	☐ MRI-based neuroimaging

Antibodies

Antibodies used	The following antibodies (vendor, catalog number) were used for Western blotting assays: DDX41 (CST, #15076), BG4 (Absolute Antibody, Ab00174), p53 (CST, #9282), cGAS (E5V3W) (CST, #79978), Phospho-Histone H2A.X (Ser139) (D7T2V) (CST, #80312), NF-κB Pathway Antibody Sampler Kit (CST #9936), RPL26 (Proteintech, #17619-1-AP), RPL7A (Proteintech, 15340-1-AP), RPS27A (Proteintech, #14946-1-AP), RPS3 (Proteintech, #11990-1-AP), RPS6 (Proteintech, #14823-1-AP), RPS19 (Proteintech, #15085-1-AP), RPS14 (Proteintech, 16683-1-AP), Ribosomal Protein S3 (D50G7) (CST, #9538S), S6 Ribosomal Protein (5G10) (CST, #2217), RPL10 (Proteintech, #72912), RPL11 (D1P5N) (Proteintech, #18163), RPL5 (Proteintech, #14568), HRP-conjugated beta actin monoclonal antibody (Proteintech, #HRP-66009), anti-rabbit IgG, HRP-linked antibody (CST, #7074), and anti-mouse IgG, HRP-linked antibody (CST, #7076). For flow cytometry assays, the following antibodies were used: APC rat anti-mouse TER119 (BD, #561033), FITC anti-mouse CD71 (Biolegend #113805), PE rat anti-mouse CD71 (BD, #567206), PE anti-mouse IgG1 (Biolegend, #406607), PE rat anti-mouse CD45R/B220 (BD, #553089), APC rat anti-mouse CD3 (BD, #565643), Phospho-histone H2A.X (Ser139) (CR55T33), PE F(ab')2 fragment (Alexa Fluor® 488 conjugate) (CST, #4412), anti-mouse IgG (H+L), F(ab')2 fragment (Alexa Fluor® 647 conjugate) (CST, #4410), Phospho-Histone H2A.X (Ser139) monoclonal antibody (Invitrogen, 12-9865-43), anti-rabbit IgG F(ab')2 fragment (Alexa Fluor® 488 Conjugate) (CST, #4412), anti-mouse IgG (H+L) F(ab')2 fragment (Alexa Fluor® 647 Conjugate) (CST, #4410), FITC rat anti-mouse CD44 (BD, #561859), APC rat anti-CD11b (BD, #553312), BV421 rat anti-mouse Ly-6C (BD, #562727), PE rat anti-mouse Ly-6G (BD, #561104), FITC rat anti-mouse Ly-6A/E (BD, #557405), and APC rat anti-mouse CD117 (BD, #553356).
Validation	Each antibody was validated by the manufacturer. Validation data and protocols are available on the manufacturer's website.

Eukaryotic cell lines

Policy information about cell lines and Sex and Gender in Research

Cell line source(s)	HEK293T cells: ATCC (CRL-1573)
Authentication	The cell line was authenticated by short tandem repeat (STR) profiling by ATCC. No additional authentication was performed in-house.
Mycoplasma contamination	Negative
Commonly misidentified lines (See ICLAC register)	HEK293T is not listed as a misidentified cell line in the ICLAC (International Cell Line Authentication Committee) register. However, due to extensive subcloning (e.g., 293FT), care was taken to use the parental HEK293T line as verified by the supplier.

Animals and other research organisms

Policy information about studies involving animals; ARRIVE guidelines recommended for reporting animal research, and Sex and Gender in Research

Laboratory animals	mouse
Wild animals	NA
Reporting on sex	All findings apply to both male and female animals.
Field-collected samples	NA
Ethics oversight	All animal procedures were reviewed and approved by the Institutional Animal Care and Use Committee (IACUC) at Northwestern University.

Note that full information on the approval of the study protocol must also be provided in the manuscript.

Plants

Seed stocks | NA

Novel plant genotypes | NA

Authentication | NA

Flow Cytometry

Plots

Confirm that:

☒ The axis labels state the marker and fluorochrome used (e.g. CD4-FITC).

☒ The axis scales are clearly visible. Include numbers along axes only for bottom left plot of group (a 'group' is an analysis of identical markers).

☒ All plots are contour plots with outliers or pseudocolor plots.

☒ A numerical value for number of cells or percentage (with statistics) is provided.

Methodology

Sample preparation

For mouse bone marrow flow cytometry, mice were euthanized following ethical guidelines approved by the IACUC at Northwestern University. Femur bones were dissected to extract bone marrow cells via PBS flushing. Spleens were weighed and subsequently homogenized. These isolated single cells were labeled with antibodies for subsequent flow cytometric analysis
The quantification of G-quadruplexes was performed using the BG4 antibody with mouse IgG1 Isotype. Cells were fixed in 0.25% Glutaraldehyde/PBS for 1 hour, followed by permeabilization with 0.1% Triton-X/PBS. To remove the RNAs, cells were incubated in 0.05% Triton-X/PBS with 3% bovine serum albumin (BSA) and 10 µg/ml RNaseA for 30 minutes. Subsequently, they were incubated in PBS containing 0.05% Triton-X, 3% BSA, and 10 µg/ml BG4 antibody for at least 30 minutes. After washing, cells were resuspended in PBS with PE-conjugated anti-mouse IgG1 antibody for 10 minutes. Finally, the cells were washed and resuspended in PBS for flow cytometry analyses.

Instrument | BD LSR Fortessa 1 Analyzer, BD FACSymphony A5-Laser Analyzer

Software | FLOWJO

Cell population abundance | 10,000 - 50,000

Gating strategy | All cells were first gated by FSC/SSC to exclude debris, followed by FSC-A/FSC-H to include only single cells. For fresh bone marrow samples, PI staining were used to exclude dead cells.

☒ Tick this box to confirm that a figure exemplifying the gating strategy is provided in the Supplementary Information.
